# Inhibitory Role of the KEAP1-NRF2 Pathway in TGFβ1-Stimulated Renal Epithelial Transition to Fibroblastic Cells: A Modulatory Effect on SMAD Signaling

**DOI:** 10.1371/journal.pone.0093265

**Published:** 2014-04-01

**Authors:** In-geun Ryoo, Hunjoo Ha, Mi-Kyoung Kwak

**Affiliations:** 1 College of Pharmacy, The Catholic University of Korea, Bucheon, Gyeonggi-do, Republic of Korea; 2 College of Pharmacy, Ewha Womans University, Seodaemun-gu, Seoul, Republic of Korea; UAE University, Faculty of Medicine & Health Sciences, United Arab Emirates

## Abstract

Transforming growth factor β1 (TGFβ1) is a potent stimulator of epithelial-to-mesenchymal transition (EMT) and has been associated with chronic kidney diseases by activating profibrotic gene expression. In this study, we investigated the role of the KEAP1-NRF2 pathway, which is a master regulator of the cellular antioxidant system, in TGFβ1-stimulated EMT gene changes using human renal tubular epithelial HK2. Treatment with TGFβ1 enhanced the levels of reactive oxygen species (ROS) and TGFβ1-stimulated EMT gene changes, including an increase in profibrotic fibronectin-1 and collagen 1A1, were diminished by the antioxidant N-acetylcysteine. In HK2, TGFβ1 suppressed NRF2 activity and thereby reduced the expression of GSH synthesizing enzyme through the elevation of ATF3 level. Therefore, the activation of NRF2 signaling with sulforaphane effectively attenuated the TGFβ1-stimulated increase in fibronectin-1 and collagen 1A1. Conversely, the TGFβ1-EMT gene changes were further enhanced by *NRF2* knockdown compared to the control cells. The relationship of NRF2 signaling and TGFβ1-EMT changes was further confirmed in a stable *KEAP1*-knockdown HK2, which is a model of pure activation of NRF2. The TGFβ1-mediated increase of collagen 1A1 and fibronectin-1 in *KEAP1* knockdown HK2 was suppressed. In particular, TGFβ1-SMAD signaling was modulated in *KEAP1* knockdown HK2: the TGFβ1-stimulated SMAD2/3 phosphorylation and SMAD transcriptional activity were repressed. Additionally, the protein level of SMAD7, an inhibitor of SMAD signaling, was elevated and the level of SMURF1, an E3 ubiquitin ligase for SMAD7 protein, was diminished in *KEAP1* knockdown HK2. Finally, the inhibition of *SMAD7* expression in *KEAP1* knockdown HK2 restored TGFβ1 response, indicating that SMURF1-SMAD7 may be a molecular signaling linking the NRF2-GSH pathway to TGFβ1-EMT changes. Collectively, these results indicate that the KEAP1-NRF2 antioxidant system can be an effective modulator of TGFβ1-stimulated renal epithelial transition to fibroblastic cells through the SMUR1-SMAD7 signaling, and further implies the beneficial role of NRF2 in chronic renal diseases.

## Introduction

The epithelial-to-mesenchymal transition (EMT), a phenotypic transition of epithelial cell, is a complex and dynamic phenomenon that is accompanied by a loss of the epithelial cell hallmarks and an acquisition of the mesenchymal characteristics [1]–[3]. Through the EMT process, epithelial cells initiate the reprogramming of gene expression and exhibit the reduced expression of epithelial markers such as claudin-1 (CLDN1) and E-cadherin (CDH-1). Conversely, the expression of mesenchymal markers such as α-smooth muscle actin (α-SMA), fibroblast-specific protein-1 (FSP-1), collagen (COL) and fibronectin (FN) increase [3]–[5]. EMT has been well established in embryonic development; in addition, extensive studies during the last few decades have revealed that EMT can occur in adult epithelial cells. Kalluri and Weinberg et al. [2] classified EMT into 3 types: (i) type 1 EMT during embryogenesis, (ii) type 2 EMT during tissue repair and fibrosis, and (iii) type 3 EMT during metastasis. Evidence of EMT *in vitro* has been obtained in a renal tubular epithelial cell system with various EMT stimulators such as transforming growth factor β (TGFβ), connective tissue growth factor (CTGF), interleukin-1, and angiotensin II (ATII) [5]–[9]. In particular, TGFβ1, as a sole factor, can induce EMT gene changes in renal tubular cells and stimulates production of extracellular matrix (ECM) proteins such as collagen and fibronectin [10]–[13]. Consistent with these *in vitro* results, the involvement of EMT in renal pathology has been demonstrated in multiple *in vivo* studies. In biopsy samples from patients with chronic kidney diseases (CKD), the tubular expression of epithelial markers disappeared, while the expression of the mesenchymal marker FSP-1 increased [14]–[16]. The expression levels of EMT markers are well correlated with renal dysfunction [15].

TGFβ1 is a cytokine with multiple functions and is widely accepted as a profibrogenic factor, which leads to the accumulation of ECM [7], [8]. During fibrotic pathology, the level of TGFβ1 is increased and this in turn can induce EMT change and recruit inflammatory cells and fibroblasts to stimulate the production of cytokines and further ECM. It is apparent that TGFβ1-induced EMT is mediated primarily through SMAD signaling [17], [18]. As the canonical pathway, receptor activation by TGFβ1 leads to the phosphorylation of SMAD2 and SMAD3, and triggers complex formation of p-SMAD2/SMAD3 with SMAD4. This complex is subsequently translocated into the nucleus and transactivates the expression of target genes encoding ECM, metalloproteases, CTGF, and Snail, which is a transcription factor regulating EMT genes. SMAD7, the inhibitory SMAD, antagonizes TGFβ1 signaling through negative-feedback actions [19]. In addition to the canonical pathway, TGFβ1 activates other signaling molecules, including mitogen-activated protein kinases (MAPKs) and phosphoinositide 3-kinase (PI3K)/AKT [17], [20]. Reactive oxygen species (ROS) have been proposed as potent contributors to TGFβ1-mediated pathology [21], [22]. The activation of NADPH oxidase (NOX) has been suggested as a cause of the TGFβ1-mediated increase of ROS in renal myofibroblasts and endothelial cells [23], [24]. Therefore, the inhibition of NOX4 using small interfering RNA (siRNA) could block α-SMA expression by TGFβ1 in rat kidney fibroblasts [23]. Whereas, in certain cases, TGFβ1 can induce EMT in a receptor-independent manner: in human glomerular mesangial cells, TGFβ1 activates the PI3K/AKT pathway and this triggers SMAD3 phosphorylation and a further increase in collagen expression [25].

Nuclear factor (erythroid-derived 2)-like 2 (NRF2) plays a crucial role in the regulation of both the basal and inducible expression of an array of genes encoding: (i) direct ROS scavenging enzymes, such as superoxide dismutase (SOD) and glutathione (GSH) peroxidase (GPx), (ii) thiols and their generating enzymes, (iii) electrophile detoxifying enzymes, iv) stress response proteins such as heme oxygenase-1, and (v) molecular chaperons and proteasomes [26]–[28]. In particular, the expression of the catalytic and modulatory subunit of γ-glutamate cysteine ligase (GCL), a GSH biosynthesis enzyme, and GSH reductase (GSR) is upregulated by NRF2 through the antioxidant response element (ARE) located in their promoters [29], [30]. NRF2 activity is mainly controlled by the cytoplasmic protein inhibitor Kelch-like ECH-associated protein 1 (KEAP1) [31]. Under normal conditions, NRF2 is tethered to the KEAP1 protein in the cytoplasm, resulting in the blockade of NRF2 nuclear translocation and proteasomal degradation through the action of Cullin 3-based E3 ligase [32], [33]. Under oxidizing conditions, conformational changes of KEAP1 result in the liberation of NRF2 and the transactivation of ARE-bearing genes by forming a heterodimer with small MAF proteins [34], [35]. In particular, other bZIP proteins such as BACH1 and ATF3 can negatively regulate NRF2 activity by inhibiting ARE transactivation [36], [37]. Due to coordinated elevation of antioxidant proteins, NRF2 activating small molecules such as sulforaphane (SFN) exhibit protective effects against oxidative stress-associated damage [38]–[40].

Recent several reports showed that NRF2 signaling exhibits beneficial effects on CKD. The treatment of animals with NRF2 inducers, including SFN and curcumin, alleviated renal damage in a model of diabetic nephropathy and nephrectomy [41], [42]. In a rat tubular epithelial cell system, the NRF2 activating dimethylfumarate treatment as well as forced expression of NRF2 attenuated TGFβ1-induced SMAD3 phosphorylation and collagen expression [43]. In addition, in the kidneys from experimental animal models of CKD, severe oxidative stress and inflammation have been explained by impaired NRF2 activity and reduced target gene expression [44], [45]. In the current study, we have investigated the role of the KEAP1-NRF2 antioxidant system in TGFβ1-induced EMT changes using human renal tubular epithelial HK2 cells. The stable HK2 cell lines with genetic alterations in *KEAP1* or *NRF2* expression were established and TGFβ1 response was assessed to ask the involvement of NRF2 signaling in EMT change. At the same time, as a pharmacological approach, the effect of the NRF2-activating small molecule SFN was investigated in TGFβ1-treated renal epithelial cells. Using these different experimental models, we show that the NRF2-GSH signaling can be an effective modulator in TGFβ1-induced EMT changes. In particular, we provide evidence that SMURF1-SMAD7 could be target molecules linking the NRF2-GSH pathway to SMAD signaling inhibition.

## Materials and Methods

### Materials

Recombinant human TGFβ1 was purchased from Cell Signaling Technology (Beverly, MA, USA) and was solubilized in sterile 20 mM citrate (pH.3.0). SFN was obtained from Sigma-Aldrich (Saint Louis, MO, USA). Antibodies recognizing pSMAD2, pSMAD3, SMAD7, and SMURF1 were from Cell Signaling Technology. β-Tubulin and ATF3 antibodies were purchased from Santa Cruz Biotechnology (Santa Cruz, CA, USA). The luciferase reporter plasmid containing the ARE was a gift from Dr. Nobuano Wakabayashi (University of Pittsburg, PA, USA) [34]. The reporter plasmid containing the human SMAD-responsive element (SRE) was obtained from Addgene (Cambridge, MA, USA). The lentiviral expression plasmids for human *NRF2* and *KEAP1* short hairpin RNA (shRNA), lentiviral packaging mix, hexadimethrine bromide, and puromycin were from Sigma-Aldrich. Reagents for GSH measurement, including NADPH, GSR, and GSH, were obtained from Sigma-Aldrich.

### Cell culture

The normal human kidney tubular epithelial HK2 cell line was obtained from the American Type Culture Collection (Manassas, MD, USA). HK2 cells were maintained in Dulbecco's Modified Eagle's Medium (DMEM):Nutrient Mixture F-12 (HyClone, Logan, UT, USA) with 10% fetal bovine serum (FBS; HyClone) and penicillin/streptomycin (HyClone). The cells were grown at 37°C in a humidified 5% CO_2_ atmosphere.

### Production of shRNA lentiviral particles

Lentiviral particles were produced in HEK 293T cells following the transfection of the cells with the relevant shRNA expression plasmid (Sigma-Aldrich) and lentiviral packaging mix (Sigma Aldrich) as described previously [46]. Briefly, HEK 293T cells were seeded in 60-mm plates at a density of 7.0×10^5^ cells/well. The next day, the medium was replaced with Opti-MEM (Invitrogen, Carlsbad, CA, USA) and, subsequently, 1.5 μg pLKO.1-NRF2 shRNA, (5′-CCGGGCTCCTACTGTGATGTGAAATCTCGAGATTTCACATCACAGTAGGA-3′) or pLKO.1-KEAP1 shRNA (5′-CCGGGTGGCGAATGATCACAGCAATCTCGAGATTGCTGTGATCATTCGCCACTTTTTTG-3′) and the packaging mix were transfected into the cells by using Lipofectamine 2000 (Invitrogen). As a nonspecific RNA, the pLKO.1-scrambled (sc) RNA plasmid was transfected in the control group. On the second day, the medium containing the transfection complex was removed. The medium containing lentiviral particles was harvested after 4 days.

### Establishment of *NRF2*- or *KEAP1*-knockdown cells

HK2 cells in 6-well plates were transduced with lentiviral particles containing the nonspecific pLKO.1-scRNA, pLKO.1-*NRF2* shRNA, or pLKO.1-*KEAP1* shRNA in the presence of 8 μg/ml hexadimethrine bromide (Sigma-Aldrich). Transduction was continued for 48 h followed by a 24 h-recovery in complete medium. For the selection of stable transgene-expressing cells, puromycin (2 μg/ml) selection was performed for up to 4 weeks.

### SMAD7 siRNA transfection

Pre-designed and pre-annealed SMAD7 siRNA (100 nM) and nonspecific scrambled siRNA (100 nM) comprising 19-bp sequence with 3′-dT overhangs were purchased from Bioneer (Daejeon, South Korea). HK2 cells in 6-well plates were grown for 24 h in the absence of antibiotics and transfected with nonspecific or SMAD7-specific siRNA using a Lipofectamine reagent (Invitrogen). RNAs were extracted after the recovery of 24 h in the complete medium.

### Total RNA extraction and RT-PCR analysis

Total RNA was isolated from the cells using the TRIzol reagent (Invitrogen). For the synthesis of cDNA, reverse transcriptase (RT) reactions were performed by incubating 200 ng of total RNA with a reaction mixture containing 0.5 μg/μl oligo dT_12–18_ and 200 U/μl Moloney murine leukemia virus RT (Invitrogen). Real-time RT-polymerase chain reaction (PCR) analysis for relative quantifications was carried out using a Roche LightCycler (Mannheim, Germany) with the Takara SYBR Premix ExTaq System (Otsu, Japan). Primers were synthesized by Bioneer and primer sequences for the human genes are: *NRF2* 5′-GTGCATCTGCACAGGCAACG-3′ and 5′-TGGCCATAGGGAGGAGGCTG-3′; *KEAP1*, 5′-GGAGGCTATGATGGTCACAC-3′ and 5′-AGTTCTGCTGGTCAATCTGC-3′; *α-SMA*, 5′-TGTGGCTATCCAGGCGGTGC-3′ and 5′-TCTCGGCCAGCCAGATCCAGAC-3′; *FN-1*, 5′-TCGGCGAGAGCATGACCGAT-3′ and 5′-GGCCACGCTGTTCTTGCAGT-3′; *COL1A1*, 5′-ATAGCTGAGCCCAGTATC-3′ and 5′-CATGCACGTGAGTGCTCT-3′; *CLDN1*, 5′-GGTGCAGAAGATGAGGATGGCTG-3′ and 5′-AGCCAGTGAAGAGAGCCTGACC-3′; *SNAI1*, 5′-TCTAGGCCCTGGCTGCTACAAG-3′ and 5′-ATTTCTGTGTTGGCGCAGTGTGGTC-3′; *SMAD2*, 5′-AGCAGAATACCGAAGGCAGACGG-3′ and 5′-TGACATGCTTGAGCAACGCACTG-3′; *SMAD3*, 5′-TGAACCACAGCATGGACGCAGG-3′ and 5′-GCGCTGGTTCAGCTCGTAGTAG-3′; *SMAD7*, 5′-GTCAAGAGGCTGTGTTGCTG-3′ and 5′- GACAGTCTGCAGTTGGTTTGAG-3′; *NOX1*, 5′-AGTCATCCTCGCAAGTGTGCAG-3′ and 5′-TCACAACCTTCTGCTGGGAGCG-3′; *NOX4*, 5′-CCCATGTGCCGAACACTCTTGG-3′ and 5′-TGAGGGCATTCACCAGATGGGC-3′; hypoxanthine-guanine phosphoribosyltransferase (*HPRT*), 5′-GGACTAATTATGGACAGGAC-3′ and 5′-TGCATTGTTTTGCCAGTGTC-3′. The primer sequences for the human catalytic (GCLC) and modulatory subunit (GCLM) of GCL, GSR, superoxide dismutase 1 (SOD1), and GPX 1 and 2 are described previously [47].

### Immunoblot analysis

The protein samples were separated by electrophoresis on 6–10% SDS-polyacrylamide gels and transferred to nitrocellulose membranes (Whatman, Dassel, Germany) by using a Trans-Blot Semi-Dry Cell (Bio-Rad). The membrane was blocked with 5% skimmed milk for 1 h, and then incubated with the antibodies. Following the addition of the enhanced chemiluminescence reagent (Amersham Biosciences, Buckinghamshire, UK), images were detected using a ChemiDoc System (Bio-Rad).

### Measurement of total GSH content

Cells in 6-well plates were lysed with a 5% metaphosphoric acid solution. The total GSH content was determined by measuring the changes in optical density for 4 min following the incubation of 30 μl cell lysate with 30 μl 5, 5′-dithiobis-(2-nitrobenzoic acid), GSR and β-NADPH. The protein concentration was determined using a BCA Protein Assay Kit (Pierce, IL, USA).

### Measurement of ROS

Cellular ROS levels were determined using the fluorescent probe 5-(and-6)-carboxy-2′,7′-dichlorofluorescein diacetate (carboxy-H_2_DCFDA). Briefly, cells in 96-well plates were treated with TGFβ1 for indicated times. Then, the cells were incubated with 25 μM carboxy-H_2_DCFDA for 30 min at 37°C and fluorescence intensity was measured with a plate reader (Perkin Elmer Victor X3, Waltham, MA, USA) at 485 nm excitation and 535 nm emissions.

### Measurement of luciferase activity

The cells were seeded in 24-well plates at a density of 2.0×10^4^ cells/well and grown overnight. The next day, the transfection complex containing 0.5 μg of ARE-luciferase plasmid or SRE-luciferase plasmid along with 0.05 μg of pRLtk control plasmid (Promega) and the transfection reagent (WelGENE Inc., Daegu, South Korea) were added to each well. After 18 h, the transfection complex was removed and the cells were incubated in complete medium for 24 h. The cells were then lysed, and Renilla and firefly luciferase levels were measured using the Dual Luciferase Assay System (Promega) with a luminometer (Turner Designs, Sunnyvale, CA, USA).

### Statistical Analyses

Statistical significance was analyzed using Student's *t*-test or a one-way analysis of variance (one-way ANOVA) followed by the Student Newman–Keuls test for multiple comparisons using Prism software (GraphPad Prism, La Jolla, CA, USA).

## Results

### Effect of TGFβ1-ROS on EMT gene changes in human renal tubular epithelial HK2

**Figure 1:**
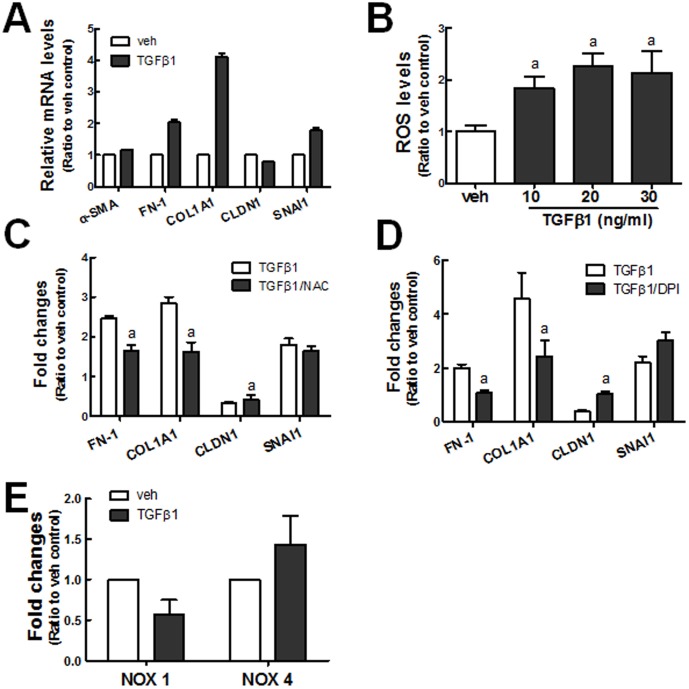
TGFβ1-induced EMT gene changes are associated with oxidative stress. (A) HK2 cells were incubated with vehicle (veh: 20 mM citrate, pH 3.0) or TGFβ1 (10 ng/ml) for 48 h, and the transcript levels of *α-SMA, FN-1, COL1A1, CLDN1*, and *SNAI1* were monitored using real-time RT-PCR for relative quantification. The values are ratios with respect to each veh control and are the means ± SE of 3 experiments. (B) Cellular ROS levels were monitored in HK2 cells treated with TGFβ1 (10, 20, and 30 ng/ml) for 48 h. The values are ratios with respect to the veh control and are the means ± standard error (SE) of 3–4 experiments. ^a^P<0.05 compared with the veh control. (C) The effect of NAC on TGFβ1-induced gene changes was examined in HK2 cells. The transcript levels for *FN-1, COL1A1, CLDN1*, and *SNAI1* were estimated following incubation with TGFβ1 or NAC (5 mM) plus TGFβ1 for 48 h. (D) HK2 cells were pre-incubated with DPI (1 μM) for 24 h prior to incubation with TGFβ1 (48 h), and the transcript levels of *FN-1, COL1A1, SNAI1*, and *CLDN1* were measured using real-time RT-PCR. (E) The transcript levels for *NOX1* and *NOX4* were determined in cells following TGFβ1 incubation (10 ng/ml, 48 h). The values are ratios with respect to each veh control and are the means ± SE of 3 experiments. ^a^P<0.05 compared with the TGFβ1 group.

In order to test whether TGFβ1 induces EMT gene changes in human renal tubular epithelial HK2, cells were incubated with TGFβ1 (10 ng/ml) for 48 h, and the expression of EMT genes such as *α-SMA, FN-1, COL1A1, CLDN1*, and *SNAI1* was investigated using real-time RT-PCR analysis. The transcript levels of ECM genes *COL1A1* (4-fold) and *FN-1* (2-fold) were elevated, and CLDN1, an epithelial marker, was decreased following TGFβ1 treatment (Fig. 1A). In addition, the level of SNAI1, which encodes Snail transcription factor, was increased. These indicate that the incubation of HK2 with TGFβ1 can induce EMT-like changes with respect to gene expression and morphological changes (Data not shown). Since several lines of evidence show that ROS may be associated with TGFβ1-mediated EMT and further fibrogenesis [22], [48], [49], we estimated the levels of cellular ROS. The levels of ROS were significantly increased by TGFβ1 incubation for 48 h, and 10 ng/ml TGFβ1 elevated the levels of ROS by up to 2-fold (Fig. 1B). When cells were pre-incubated with GSH precursor N-acetylcysteine (NAC, 5 mM) for 24 h prior to TGFβ1 treatment the increases in *FN-1* and *COL1A1* expression and the decrease in CLDN1 expression were alleviated (Fig. 1C). Whereas, the SNAI1 increase was not affected by NAC treatment. Similarly, in cells treated with diphenyleneiodonium (DPI), a NOX inhibitor, the TGFβ1-stimulated *COL1A1* and *FN-1* expression was significantly attenuated, and CLDN1 decrease was prevented (Fig. 1D). In agreement with the effect of DPI, we observed that the expression level of NOX4, which is known to play a critical role in TGFβ1-mediated ROS increase, was marginally elevated by TGFβ1 (Fig. 1E). These data indicate that TGFβ1-induced EMT gene changes in human renal tubular epithelial cells are closely related with the increase in the levels of ROS.

### Repressed GSH content in TGFβ1-treated HK2 is associated with NRF2

**Figure 2:**
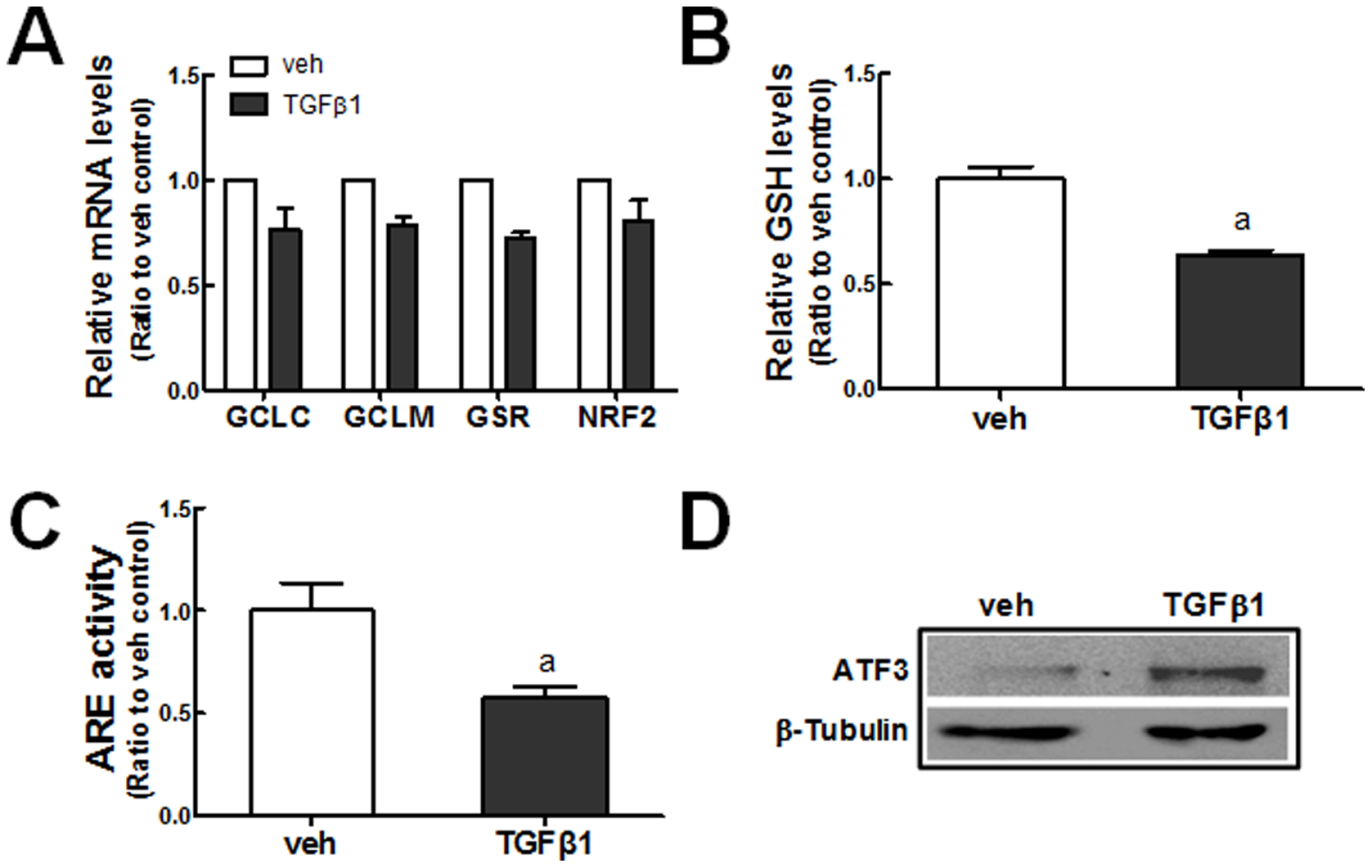
Suppression of NRF2-GSH signaling in TGFβ1-treated HK2. (A) Transcript levels of *GCLC, GCLM, GSR*, and *NRF2* were measured in vehicle (veh) or TGFβ1 (10 ng/ml, 48 h)-treated HK2 cells using real-time RT-PCR for relative quantification. The values are ratios with respect to the veh control and are the means ± SE of 3 experiments. (B) Levels of total cellular GSH content were measured in HK2 cells treated with vehicle (veh) or TGFβ1 (10 ng/ml) for 48 h. (C) ARE-driven luciferase activity was monitored in HK2 cells following incubation with TGFβ1 (10 ng/ml, 8 h). ^a^P<0.05 compared with the veh control group. (D) Protein levels of ATF3 were determined in HK2 cells following incubation with TGFβ1 (10 ng/ml, 8 h). Total cell lysates were used for immunoblot analysis of ATF3.

GSH plays a critical role in the removal of cellular ROS. Since previous reports showed that TGFβ1 represses the expression of GCL in mammary epithelial cells, elevated ROS in TGFβ1-treated HK2 may be related with altered GSH biosynthesis [50], [51]. A relative quantification of transcript levels for GCLC, GCLM, and GSR showed that these GSH biosynthesis and recycling enzyme levels were decreased by 20–30% following TGFβ1 treatment (Fig. 2A). Consistent with these, the treatment of HK2 cells with TGFβ1 significantly decreased the total levels of GSH (Fig. 2B). Reduced GSH genes can be associated with NRF2 activity: ARE-driven luciferase activity, which reflects NRF2 transcription activity, was significantly reduced (∼42%) after TGFβ1 treatment for 8 h (Fig. 2C). Whereas, protein level of NRF2 was not affected by TGFβ1 treatment (Data not shown), and the level of ATF3 protein, a repressive partner of NRF2, was elevated in TGFβ1-treated cells (Fig. 2D). These results draw a conclusion that TGFβ1 leads to the suppression of NRF2 signaling through ATF3 expression as an early event, and this, in turn, reduces the expression of GSH-generating enzymes and further increases ROS levels.

### Protective effect of SFN on TGFβ1-induced EMT gene changes

**Figure 3:**
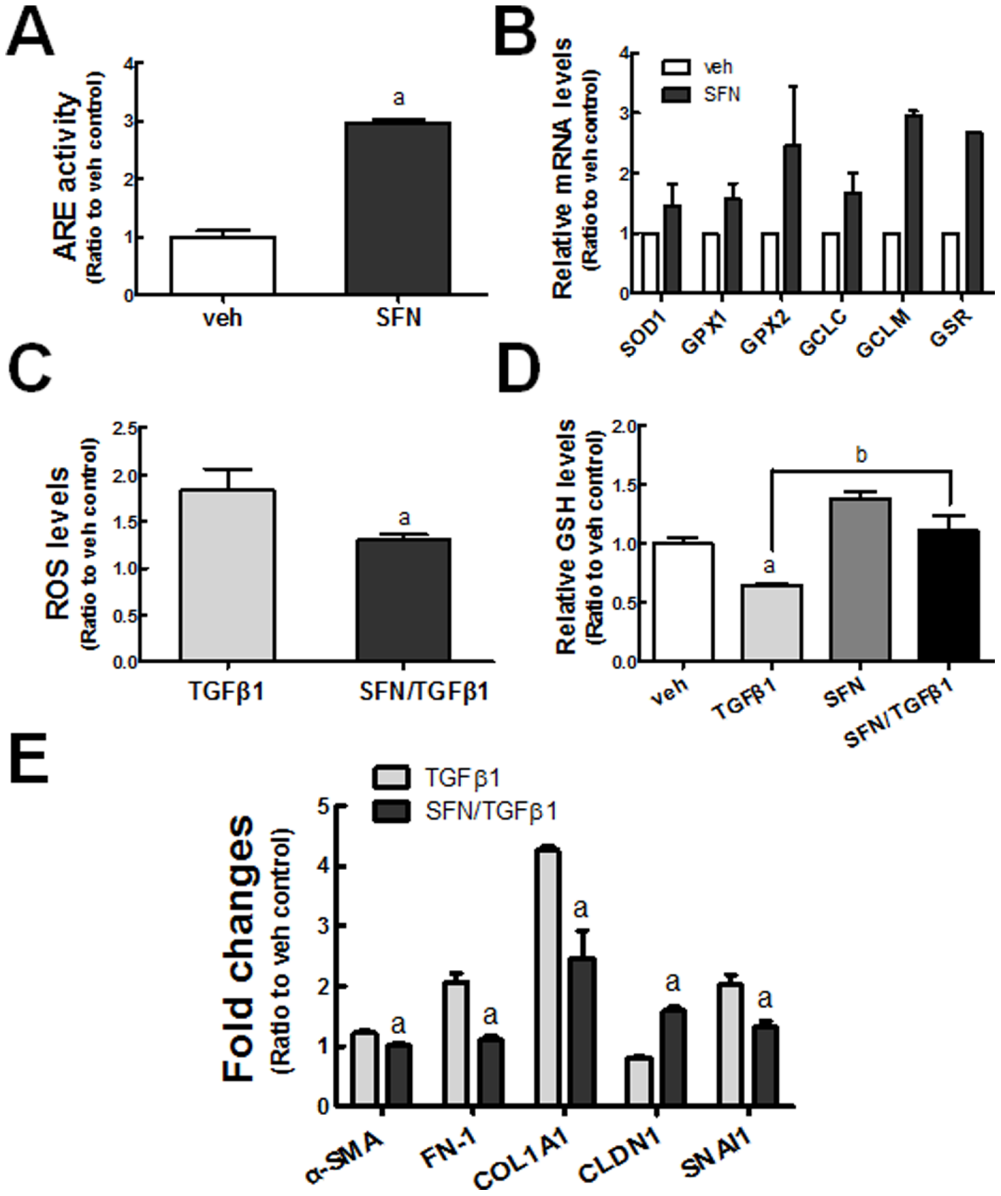
Effect of NRF2 activating SFN on TGFβ1-induced EMT gene changes. (A) ARE-driven luciferase activity was monitored in HK2 cells following incubation with vehicle (veh; DMSO) or SFN (5 μM) for 24 h. ^a^P<0.05 compared with the veh control group. (B) Transcript levels for *SOD1, GPX1, GPX2, GCLC, GCLM*, and *GSR* were determined in HK2 cells following incubation with 5 μM SFN for 24 h. (C) HK2 cells were incubated with veh (DMSO) or SFN (5 μM, 24 h) prior to incubation with TGFβ1 (48 h), cellular ROS levels were assessed. ^a^P<0.05 compared with the TGFβ1 group. (D) HK2 cells were incubated with veh (DMSO) or SFN (5 μM, 24 h) prior to incubation with TGFβ1 (48 h), and the total cellular GSH content was determined. ^a^P<0.05 compared with the veh control group. ^b^P<0.05 compared with the TGFβ1 group. (E) HK2 cells were incubated with veh (DMSO) or SFN (5 μM, 24 h) prior to incubation with TGFβ1 (48 h), and the transcript levels of EMT genes (*α-SMA, FN-1, COL1A1, CLDN1*, and *SNAI1*) were determined using real-time RT-PCR analysis. ^a^P<0.05 compared with the TGFβ1 group. All indicated values are ratios with respect to the veh control and are the means ± SE of 3–4 experiments.

SFN has been studied extensively as an effective activator of NRF2 in various experimental models. In HK2 cells, ARE-driven luciferase activity was increased by 3-fold following SFN incubation (5 μM) for 24 h (Fig. 3A). The expression of NRF2 target genes was elevated by SFN treatment: SFN increased GCLC (1.7-fold), GCLM (3.1-fold), and GSR (2.7-fold) levels compared to the vehicle (dimethyl sulfoxide, DMSO)-treated group (Fig. 3B). In line with the elevated expression of GSH-generating enzymes, SFN treatment increased the total cellular levels of GSH by up to 38% in HK2 cells (Fig. 3D), and reduced the TGFβ1-induced increase of ROS levels by 32% (Fig. 3C). As a consequence, TGFβ1-mediated reduction of GSH content was significantly alleviated in the SFN-pretreated group (Fig. 3D). On the basis of these observations, we next examined whether SFN has a modulatory effect on TGFβ1-EMT gene changes. Results showed that the transcript level of the epithelial marker CLDN1 was increased by 2-fold following SFN pretreatment (Fig. 3E). Conversely, TGFβ1-stimulated *FN-1, COL1A1, α-SMA*, and *SNAI1* levels were attenuated by SFN pretreatment. These results suggest that SFN can ameliorate TGFβ1-induced EMT gene changes by diminishing the increase of ROS levels, implying a role of NRF2 in TGFβ1 effect.

### Aggravation of TGFβ1-induced EMT gene changes in *NRF2*-knockdown HK2

**Figure 4:**
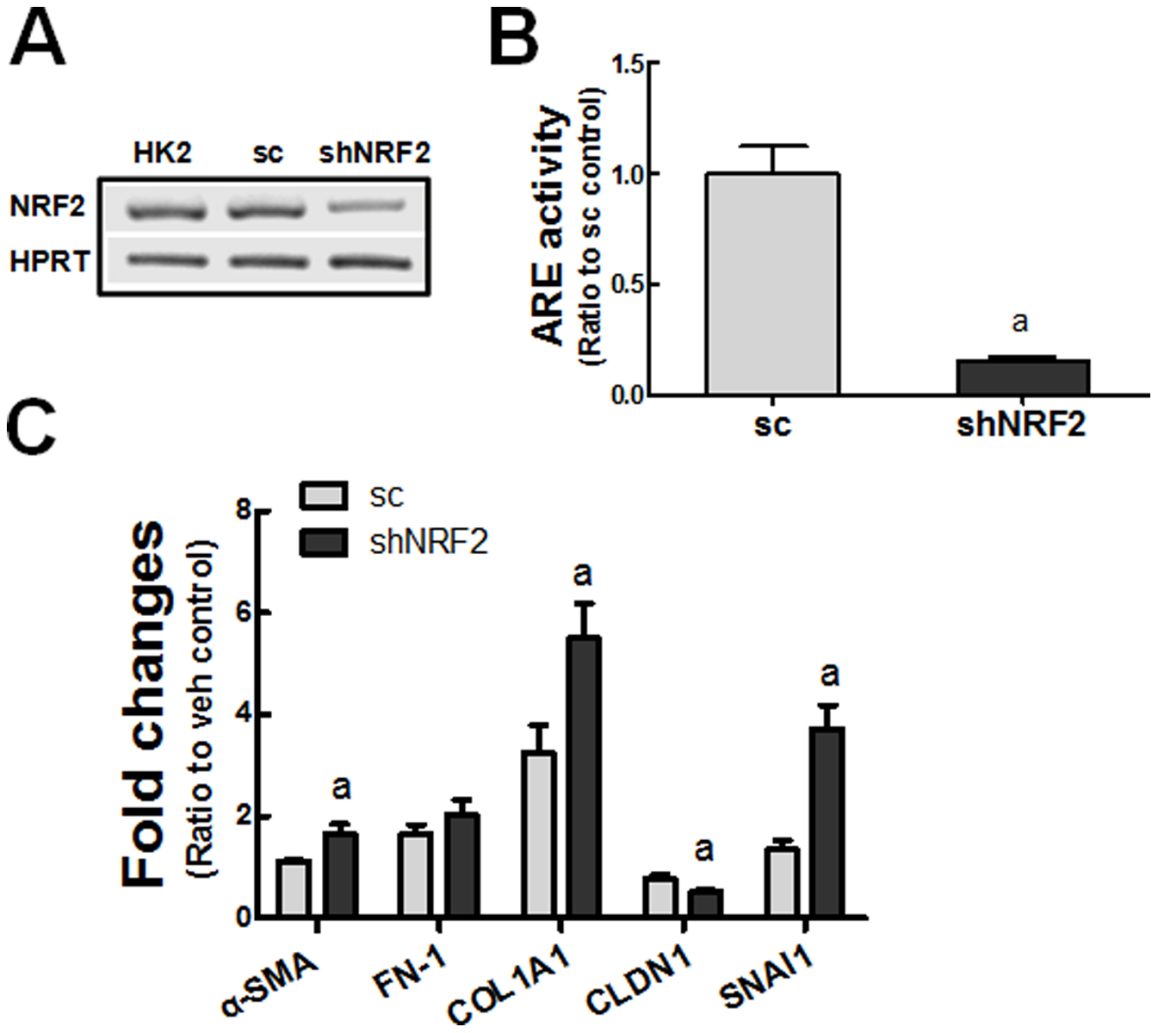
Enhanced EMT gene changes in *NRF2*-knockdown HK2 cells. (A) Transcript levels of *NRF2* in unmodified HK2 cells, nonspecific scRNA-expressing HK2 (sc) cells, and NRF2 shRNA-expressing HK2 (shNRF2) cells. (B) ARE-driven luciferase activity was measured in the sc and shNRF2 HK2 cells. ^a^P<0.05 compared with the sc cells. (C) The sc and shNRF2 HK2 cells were incubated with TGFβ1 (10 ng/ml) for 48 h and the transcript levels of EMT genes were determined. The values are ratios with respect to each vehicle (veh)-treated sc control and are the means ± SE of 3 experiments. ^a^P<0.05 compared with the TGFβ1-treated sc cells.

We established *NRF2*-knockdown HK2 (shNRF2) cells and examined EMT gene changes following TGFβ1. In these shNRF2 cells, the transcript level of NRF2 was reduced to 30% of the level in the sc cells (Fig. 4A), and NRF2 transcription activity monitored by ARE-driven luciferase was decreased by 85% compared to the sc cells (Fig. 4B). The transcript levels for TGFβ1-stimulated *α-SMA, COL1A1*, and *SNAI1* were significantly higher in shNRF2 cells than those in the sc cells (Fig. 4C). At the same time, the degree of *CLDN1* reduction was higher in TGFβ1-treated shNRF2 cells. These observations support direct involvement of NRF2 in the TGFβ1-EMT gene changes.

### Attenuation of EMT gene changes by *KEAP1*-knockdown HK2

**Figure 5:**
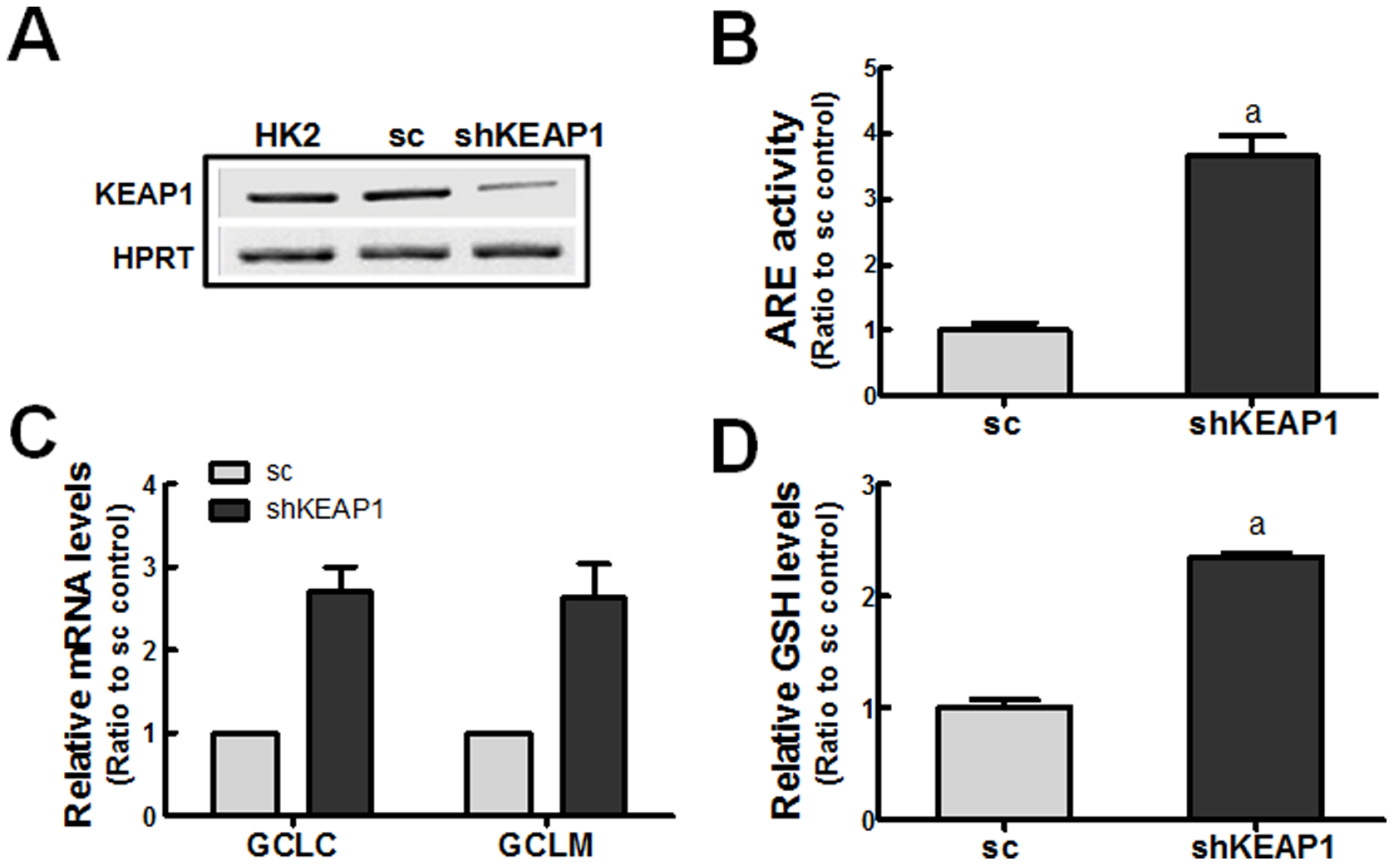
Elevated GSH levels in *KEAP1*- knockdown HK2. (A) Transcript levels of *KEAP1* in HK2 cells with stable expression of scRNA (sc) or *KEAP1* shRNA (shKEAP1). (B) ARE-driven luciferase activity was monitored in the sc and shKEAP1 HK2 cells. (C) Transcript levels for *GCLC* and *GCLM* in the scRNA (sc) and *KEAP1* shRNA (shKEAP1) HK2 cells. (D) Total GSH contents in sc and shKEAP1 HK2 cells were determined. The values are ratios with respect to the sc control and are the means ± SE of 3–4 experiments. ^a^P<0.05 compared with the sc group.

**Figure 6:**
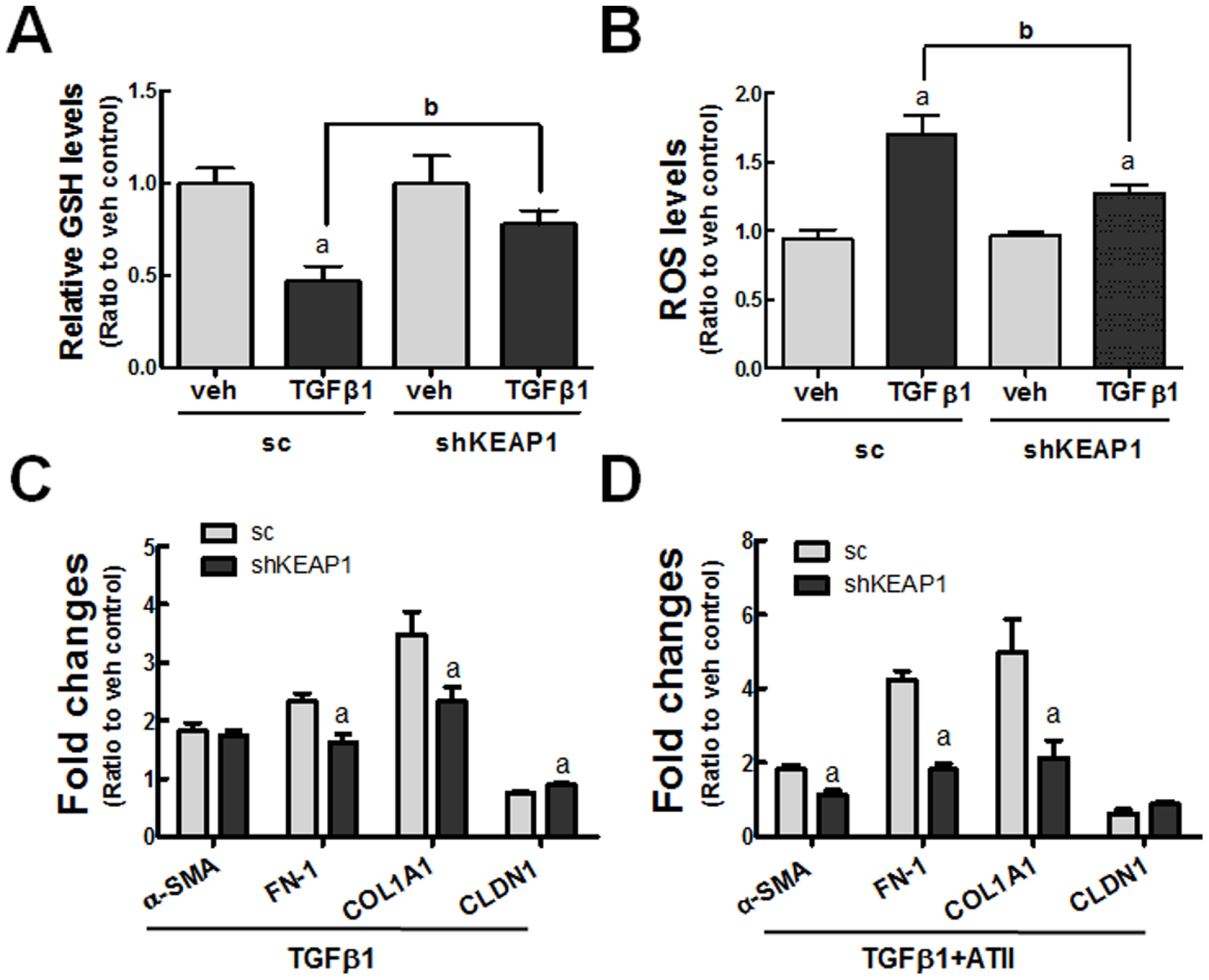
Suppressed EMT gene changes in *KEAP1*-knockdown HK2 cells. (A) The sc and shKEAP1 HK2 cells were incubated with vehicle or TGFβ1 (10 ng/ml) for 48 h, and total cellular GSH contents was determined. (B) The sc and shKEAP1 HK2 cells were incubated with TGFβ1 (48 h), and cellular ROS levels were monitored using carboxy-H_2_DCFDA. ^a^P<0.05 compared with each veh-treated group. ^b^P<0.05 compared with the TGFβ1-treated sc group. (C) HK2 cells (sc and shKEAP1) were treated with TGFβ1 (10 ng/ml) for 48 h and the transcript levels of EMT genes were assessed using real-time RT-PCR analysis. (D) HK2 cells (sc and shKEAP1) were incubated with ATII (4 μM) and TGFβ1 (10 ng/ml) for 48 h, and EMT gene changes were monitored. ^a^P<0.05 compared with the TGFβ1-treated sc group. All of the indicated values are ratios with respect to the vehicle (veh) control and are the means ± SE of 3–4 experiments.

In an attempt to confirm the involvement of NRF2 in the TGFβ1-induced EMT gene changes, we established a *KEAP1*-knockdown (shKEAP1) HK2 cell line as a pure genetic activation model of NRF2 (Fig. 5A). Accordantly, ARE-luciferase activity was elevated by 3.7-fold in the shKEAP1 cells (Fig. 5B). In *KEAP1* knockdown cells, the expression levels of *GCLC* and *GCLM* were 2.7-fold higher (Fig. 5C), and the total levels of GSH were 2.3-fold higher than those in the sc cells (Fig. 5D). These show that *KEAP1* knockdown in HK2 effectively elevates NRF2 activity. In further monitoring of TGFβ1-mediated changes, GSH depletion and ROS increase were significantly alleviated in shKEAP1 cells compared to the sc cells (Fig. 6A and 6B). As a consequence, the transcript levels of TGFβ1-stimulated *FN-1* and *COL1A1* were attenuated and the level of CLDN1 mRNA was elevated in the shKEAP1 cells when compared to the sc control cells (Fig. 6C). The resistance of *KEAP1* knockdown HK2 to EMT gene changes was also observed in cells co-incubated with TGFβ1 and ATII (4 μM), one of the stimulating factors of EMT in renal epithelial cells [52]: the levels of *α-SMA, COL1A1*, and *FN-1* were elevated by 1.8-, 4.7-, and 4.2-fold by TGFβ1 and ATII in the control cells, respectively, while their levels were significantly lower in shKEAP1 cells (Fig. 6D). These findings indicate that TGFβ1-stimulated EMT gene expression can be diminished by the genetic activation of NRF2.

### Suppression of TGFβ1-mediated SMAD signaling in shKEAP1 cells

**Figure 7:**
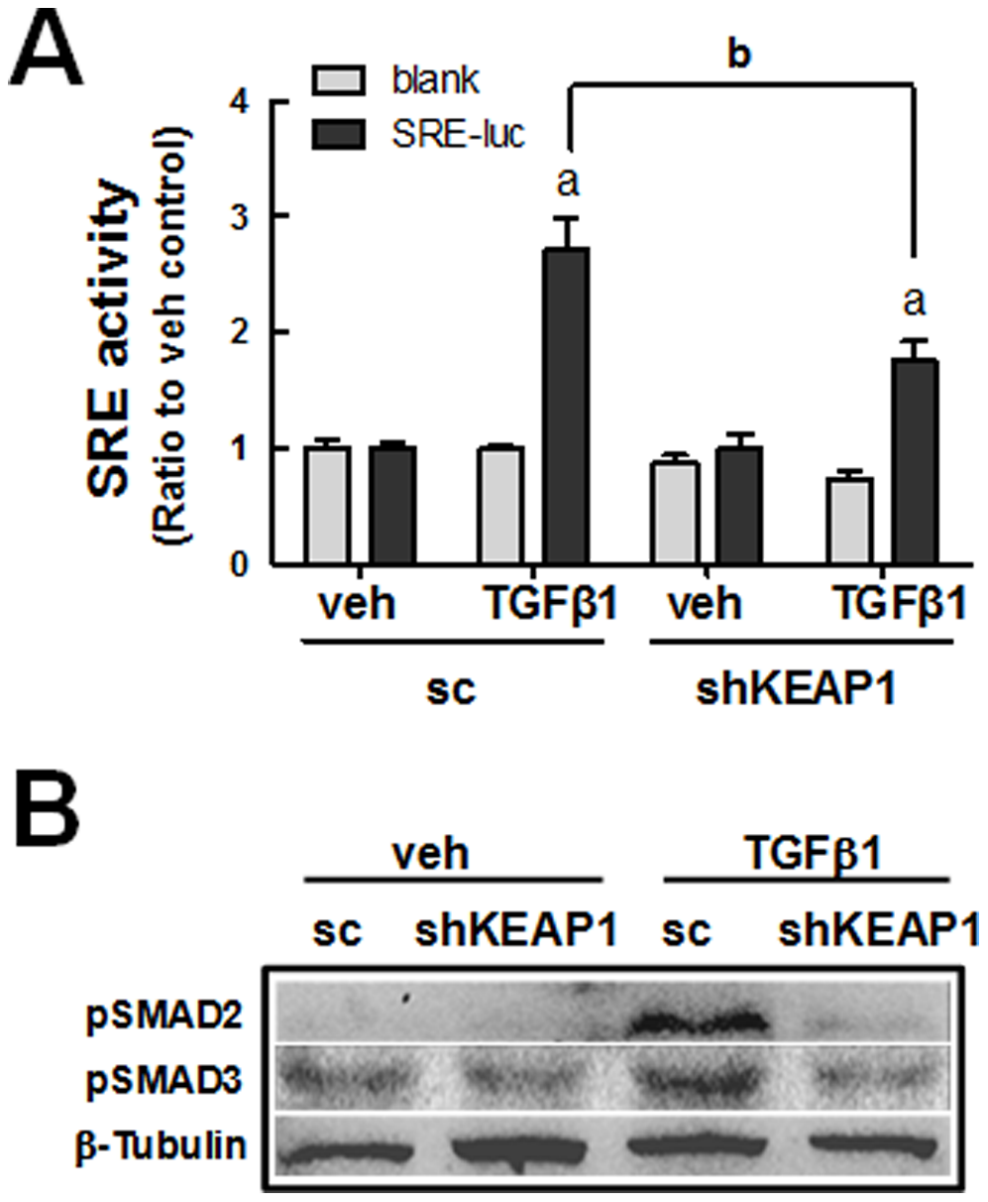
Reduced SMAD signaling in TGFβ1-treated *KEAP1* knockdown HK2. (A) Sc and shKEAP1 HK2 cells that were transiently transfected with the SMAD Response Element (SRE) reporter plasmid were incubated with veh or TGFβ1 for 18 h, and SRE-driven luciferase activity was assessed. The values are ratios with respect to each veh-treated sc control and are the means ± SE of 3–4 experiments. ^a^P<0.05 compared with each veh-treated group. ^b^P<0.05 compared with the TGFβ1-treated sc group. (B) Protein levels for phosphorylated SMAD2 and SMAD3 were assessed using immunoblot analysis following the treatment of cells with veh or TGFβ1 (10 ng/ml, 30 min) in the sc and shKEAP1 HK2 cells.

TGFβ1 binding activates receptors to stimulates SMAD2/3 phosphorylation and this, in turn, regulates the transcription of their target genes through the SRE [20]. When we performed a reporter analysis using the SRE luciferase plasmid, TGFβ1 (10 ng/ml, 18 h)-stimulated luciferase activity was reduced in shKEAP1 cells compared to the control (Fig. 7A). Accordantly, the TGFβ1-induced increase in pSMAD2 and pSMAD3 levels, which were shown after 30 min of TGFβ1 incubation, was substantially decreased in shKEAP1 cells compared to the sc cells (Fig. 7B). These observations indicate that the NRF2 pathway attenuates TGFβ1-EMT gene changes through SMAD signaling in HK2.

### Enhanced SMAD7 level in shKEAP1 cells

**Figure 8:**
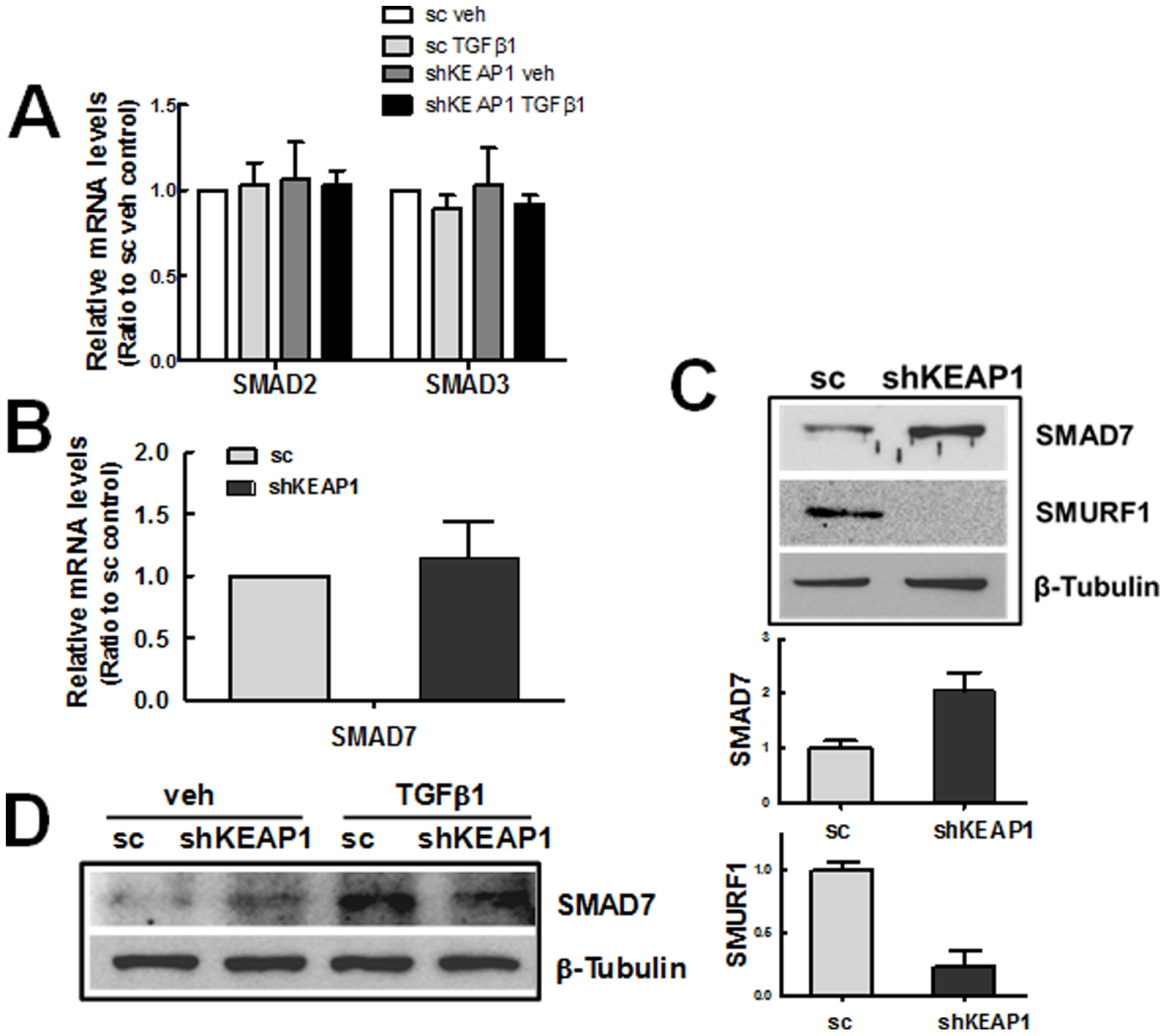
Elevation of SMAD7 level in *KEAP1*-knockdown HK2. (A) Transcript levels for *SMAD2* and *SMAD3* in the scRNA (sc) and *KEAP1* shRNA (shKEAP1) HK2 cells were determined following treatment with vehicle (veh) or TGFβ1 (24 h). (B) Levels for *SMAD7* mRNA in the sc and shKEAP1 HK2 were analyzed using real-time RT-PCR. (C) Protein levels for SMAD7 and SMURF1 were determined in the sc and shKEAP1 HK2. Bar graphs are relative values from three experiments. (D) Levels of SMAD7 protein was assessed in TGFβ1-treated (10 ng/ml, 24 h) sc and shKEAP1 HK2.

Our data suggest that elevation of GSH and amelioration of the ROS increase can lead to the inhibition of TGFβ1-EMT gene changes in *KEAP1* knockdown HK2. However, there remains a possibility that the genetic activation of NRF2 causes molecular alterations in TGFβ1-SMAD signaling, resulting in EMT gene suppression. In order to test this possibility, first, the transcript levels for SMAD2, SMAD3, and SMAD7 were assessed. These levels were not altered by *KEAP1* knockdown (Fig. 8A and 8B), whereas proteins level for SMAD7, a negative feedback regulator of SMAD signaling, was higher in the shKEAP1 cells than that in the sc control (Fig. 8C). Accordantly, we found that the protein level of SMURF1, an E3 ubiquitin ligase for proteasomal degradation of SMAD7, was substantially diminished in shKEAP1 cells. The TGFβ1-mediated SMAD7 increase did not show a significant difference between the sc control and *KEAP1* knockdown (Fig. 8D). These data indicate that altered SMURF1-SMAD7 expression can be responsible for the suppression of TGFβ1-stimulated EMT gene changes in *KEAP1* knockdown HK2.

### Restoration of TGFβ1-stimulated *FN-1* and *COL1A1* expression by SMAD7 siRNA in shKEAP1 HK2

**Figure 9:**
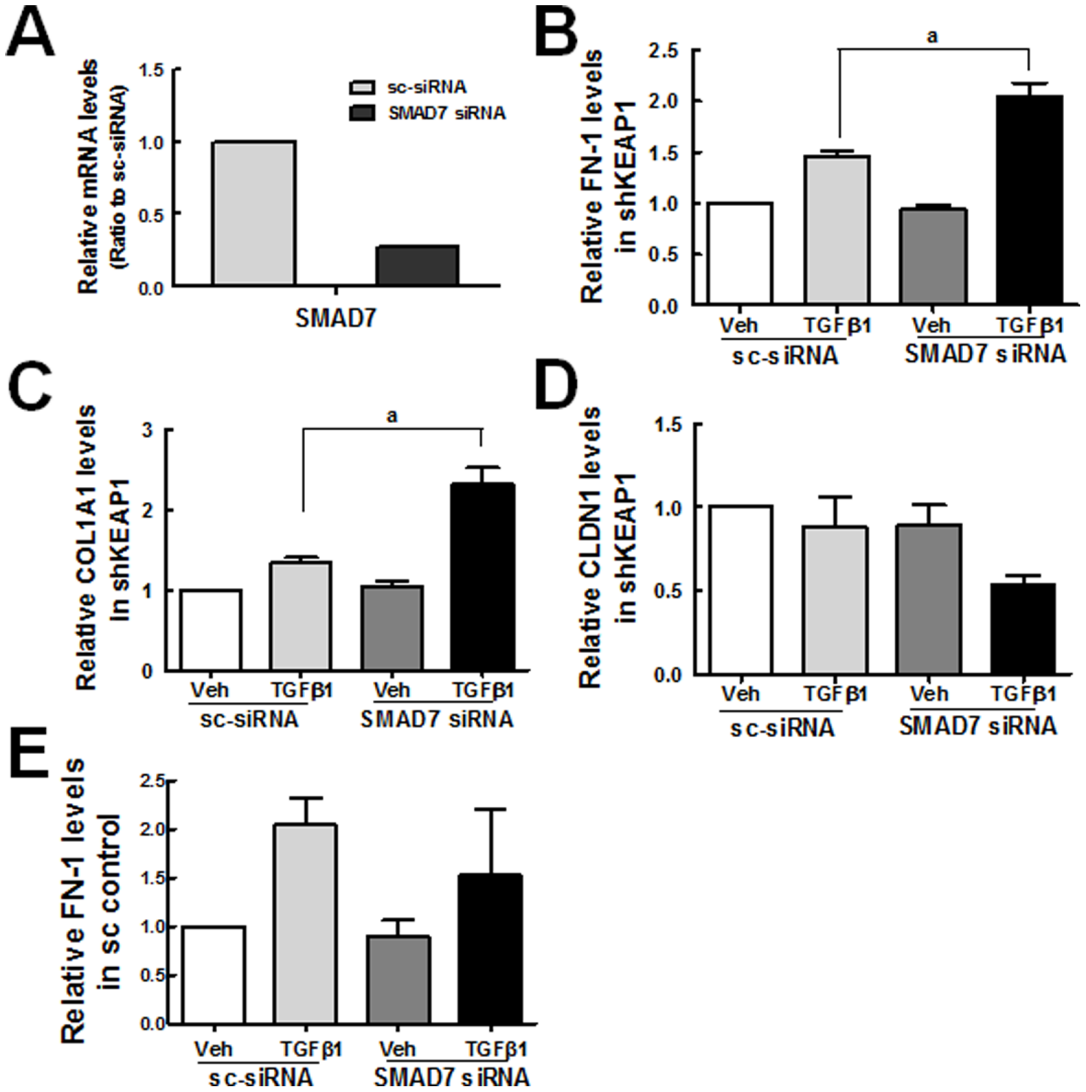
Attenuation of TGFβ1-stimulated *FN-1* and *COL1A1* expression by SMAD7 inhibition in *KEAP1* knockdown HK2. (A) HK2 cells were transfected with nonspecific siRNA or *SMAD7* specific siRNA, and transcript level for SMAD7 was estimated using real-time RT-PCR analysis. Values are ratios with respect to the nonspecific siRNA control and are the means ± SE of 3 experiments. (B-D) The *KEAP1* knockdown HK2 was transfected with nonspecific (sc-siRNA) or *SMAD7*-specific siRNA (100 nM), and transcript levels for *FN-1* (B), *COL1A1* (C), and *CLDN1* (D) were determined. (E) The sc control HK2 was transfected with nonspecific or *SMAD7*-specific siRNA, and *FN-1* level was assessed. Values are ratios with respect to the vehicle (veh)-treated sc-siRNA control and are the means ± SE of 3 experiments. ^a^P<0.05 compared with the TGFβ1-treated sc-siRNA control.

Next, to investigate the role of increased SMAD7 in diminished TGFβ1-EMT gene changes, SMAD7 was inhibited by siRNA transfection in *KEAP1* knockdown cells, and the expression of *FN-1* and *COL1A1* was assessed following TGFβ1 incubation. Firstly, after the transfection with SMAD7 siRNA, SMAD7 mRNA level reduced by 73% in *KEAP1* knockdown HK2 (Fig. 9A). Secondly, the TGFβ1-stimulated levels for *FN-1* and *COL1A1* were substantially elevated when *SAMD7* siRNA was transfected in *KEAP1* knockdown cells (Fig. 9B and 9C). In addition, the TGFβ1-mediated reduction of CLDN1 was more profound in SMAD7-inhibited shKEAP1 HK2 (Fig. 9D). While, SMAD7 inhibition in the sc control cells did not affect TGFβ1-mediated *FN-1* increase (Fig. 9E). These indicate that elevated SMAD7 may be a molecular event linking NRF2-GSH signaling to repressed TGFβ1-EMT gene changes in *KEAP1* knockdown HK2.

## Discussion

EMT, a process for the phenotypic conversion of epithelial cells to fibroblastic cells, has been hypothesized as a cellular/molecular mechanism of tissue fibrogenesis after stress and injury [2], [8], [18]. TGFβ1 is one of the strong contributors to fibrotic diseases and CKD by regulating the expression of EMT-related genes such as Snail, α-SMA, and plasmin activator inhibitor 1 (PAI-1), and by elevating ECM protein genes *COL* and *FN* [7], [9], [17]. A critical role of TGFβ1 in fibrogenesis has been supported by the observation that TGFβ1 expression is upregulated in various experimental models as well as in clinical settings [14]–[16]. In an *in vitro* experimental model with tubular epithelial cells, TGFβ1 alone can initiate and promote the EMT process. The overexpression of TGFβ1 using an active adenovirus vector increased the expression of α-SMA and type 1 *COL* in a peritoneal fibrosis animal model [53]. Conversely, an introduction of TGFβ1 siRNA delays the development of fibrosis in a unilateral ureteral obstruction (UUO) model with concomitant down-regulation of type 1 *COL* and *PAI-1* [54]. TGFβ1-mediated EMT has been associated with SMAD signaling, i.e., *smad3*-knockout mice are resistant to UUO-induced renal fibrosis and changes in the expression of EMT markers [55]. The attenuation of *smad*7 promotes EMT gene changes and renal fibrosis in animal models [56].

Like previous reports, we observed that the TGFβ1-stimulated EMT gene changes were strongly associated with oxidative stress in human renal epithelial HK2 cells. Incubation of cells with TGFβ1 resulted in an increase in ROS levels, and treatment of cells with GSH precursor NAC effectively alleviated TGFβ1-stimulated EMT gene changes (Fig, 1B and 1C). The involvement of ROS in TGFβ1-pathology has been suggested in several studies. Rhyu et al. reported that ROS generation is linked to the EMT process by TGFβ1 in a rat renal epithelial cell system and demonstrated that the inhibition of ROS generation could reduce the TGFβ1-stimulated FN secretion [48], [49]. In other report, the administration of NAC to mice attenuated the UUO-induced expression of FN [57]. As an underlying mechanism of TGFβ1-mediated oxidative stress, NOX activation has been recognized: Bondi et al. [23] showed that TGFβ1 upregulates Nox4 expression in rat kidney fibrobalsts, and the TGFβ1-stimulated α-Sma and Fn expressions are suppressed by Nox inhibitor DPI. In accordance with these observations, it is apparent that NOX-mediated ROS increase contributes to TGFβ1-EMT gene changes in HK2 (Fig. 1D) and NOX4 expression was elevated in TGFβ1-treated cells (Fig. 1E). As an additional ROS increase mechanism, TGFβ1 treatment represses NRF2 signaling in HK2. The total GSH content was decreased by TGFβ1 incubation, and this change was accompanied by a reduction in the GSH-biosynthesis enzymes GCLC and GCLM (Fig. 2A and 2B). Furthermore, the TGFβ1-incubation significantly diminished ARE reporter activity, suggesting repressed NRF2-GSH signaling by TGFβ1 (Fig. 2C). These results are in agreement with previous observations that TGFβ1 reduces the concentration of GSH in several types of cell lines including epithelial and endothelial cells through the down-regulation of GSH biosynthesis enzymes [22], [58], [59]. Bakin et al. [50] showed that TGFβ1 mediates the increase of ATF3 expression, and the ectopic expression of ATF3 was sufficient to reduce GCLC promoter activity. A recent study by Michaeloudes et al. [51] also showed that NRF2 signaling can be attenuated by TGFβ1 through an increase in ATF3 levels in cultured airway smooth muscle cells from patients. Similarly, TGFβ1 incubation elevated ATF3 protein level in HK2 (Fig. 2D), suggesting that ATF3 modulation can result in the inhibition of NRF2-GSH signaling and consequent ROS increase, which can exacerbate EMT gene changes.

On the basis of the linkage of TGFβ1-EMT gene changes and NRF2, it can be hypothesized that the KEAP1-NRF2 system can participate in the inhibition of TGFβ1 signaling for EMT gene changes in human renal tubular epithelial cells. Evidence was obtained from three experimental approaches: i) a pharmacological activation of NRF2 in HK2, ii) a genetic activation of NRF2 by *KEAP1* knockdown, and iii) a genetic knockdown of *NRF2*. By activating NRF2 signaling with SFN or *KEAP1* knockdown, the expression of *GCLC* and *GCLM* was increased, and thereby GSH depletion was alleviated with a concomitant reduction in ROS. Consequently, TGFβ1-mediated EMT gene changes, such as those in *COL1A1*, *FN-1*, *SNAI1*, and *CLDN1*, were alleviated (Fig. 3E, 6C and 6D). Conversely, in an *NRF2*-knockdown HK2 system, TGFβ1-stimulated EMT gene changes were relatively enhanced (Fig. 4C). These strongly support that the NRF2-GSH pathway is an effective modulator of phenotypic transition of renal tubular epithelial cells to fibroblastic cells by TGFβ1. Lines of evidence have shown the protective role of NRF2 activators in TGFβ1-associated fibrogenesis. The administration of solubilized CoQ10 in mice inhibited dimethylnitrosamine (DMN)-induced liver fibrosis by alleviating TGFβ1 expression [60]. The quercetin-mediated induction of HO-1 attenuated TGFβ1-stimualted collagen production in human lung fibroblasts [61]. The SFN incubation inhibited TGFβ1-induced SMAD3 phosphorylation in human hepatic stellate cell line, and mice treated with SFN were protected from hepatic fibrosis following bile duct ligation [62]. A very recent report by Artaud-Macari et al [63] demonstrated that NRF2 activation by *KEAP1* siRNA introduction induces myofibroblasts dedifferentiation in lung fibroblasts from idiopathic pulmonary fibrosis patients, and SFN treatment suppresses TGFβ1-stimulated *α-SMA* and *COL* expression in these cells. Together with these reports, our results provide direct evidence of the involvement of NRF2 signaling in TGFβ1-stimulated phenotypic conversion of epithelial cells to produce profibrotic factors by using genetic activation or inhibition systems.

It is also noteworthy that our study indicates that NRF2-mediated SMAD inhibition can be associated with enhanced SMAD7 level. In *KEAP1* knockdown HK2, the protein level of SMAD7 was elevated more than 2-fold compared to the sc control, while the transcript levels for *SMAD2, SMAD3*, and *SMAD7* were not altered. It was apparent that elevated SMAD7 level in *KEAP1* knockdown HK2 can be responsible for the inhibition of TGFβ1-stimulated *FN-1* and *COL* expression: the *SMAD7* knockdown in shKEAP1 restored TGFβ1-induced FN-1 and COL increase (Fig. 9B and 9C), whereas SMAD7 inhibition in the sc control did not affect TGFβ1 response (Fig. 9D). SMAD7 antagonizes TGFβ signaling through several mechanisms [19], [64]. As the most characterized mechanism, SMAD7 forms a complex with type I receptor of TGFβ1 and thereby inhibits SMAD2/3 phosphorylation and transactivation. Additionally, SMAD7 recruits E3 ubiquitin ligases such as SMURF1 and SMURF2 to activated type I receptor, resulting in proteasomal degradation of TGFβ1 receptor [65], [66]. In this process, SMAD7 degradation is also facilitated by the action of SMURF protein. Our data suggest that SMAD7 could be a molecule linking the NRF2-GSH pathway to SMAD signaling inhibition. As a possible cause of enhanced SMAD7 level, we observed that SMURF1 level is substantially low in the shKEAP1 cells when compared to the control (Fig. 8C). Although there is no evidence whether SMURF1 level is affected by cellular oxidative conditions, it has been documented that SMAD7 mediates the crosstalk of TGFβ1-SMAD signaling with other signaling pathways [19], [67]. The level of SMAD7 is elevated by various stimuli, including IL-1, tumor necrosis factor-α, UV irradiation, and epidermal growth factor with unidentified mechanisms. In addition, SMAD7 directly interferes with the TNF receptor associated factors (TRAF)-TAK1-TAB2/3 complex, resulting in the inhibition of NF-κB signaling, and diminishes β-catenin protein by recruiting SMURF1. These suggest a possibility that the SMURF1-SMAD7 pathway may be a component sensing cellular oxidative status and thereby linking NRF2-GSH signaling to TGFβ1-EMT gene changes in renal epithelial cells. As a supportive data, Meurer et al [68] have shown that the treatment of NAC up-regulates SMAD7 transcript level in hepatic stellate cells. Whereas, SMAD7 level was not altered in HK2 treated with SFN for 24 h (Data not shown), implying that SMAD7 change can be a long-term event accepting redox condition of cellular environment.

Collectively, this study suggests that the KEAP1-NRF2 antioxidant system can inhibit TGFβ1-stimulated EMT gene changes in renal tubular epithelial cells, and SMAD7 appears to be associated with this effect. The modulatory effect of NRF2 signaling on TGFβ1-EMT changes may provide a protective role against renal fibrogenesis, and further suggests the potential promise of small molecule NRF2 activators as a preventive intervention of CKD and fibrogenic diseases. Moreover, considering that growing evidence that shows the inhibitory effect of NRF2 in the inflammatory response, the KEAP1-NRF2 antioxidant system can be suggested as an effective target to prevent and/or control chronic renal diseases. Indeed, the triterpenoid bardoxolone, a potent activator of NRF2 as well as an anti-inflammatory small molecule, has attracted great attention as a therapeutic intervention for diabetic nephropathy [69]. Although the phase III clinical trial of bardoxolone has failed due to safety issues, our data support the beneficial effect of NRF2 signaling by showing the implication of NRF2 to epithelial transition and profibrotic gene expression.
